# Challenges and Practices in Perishable Food Supply Chain Management in Remote Indigenous Communities: A Scoping Review and Conceptual Framework for Enhancing Food Access

**DOI:** 10.3390/ijerph23010118

**Published:** 2026-01-17

**Authors:** Behnaz Gharakhani Dehsorkhi, Karima Afif, Maurice Doyon

**Affiliations:** Department of Agri-Food Economics and Consumer Sciences, Université Laval, Québec, QC G1V 0A6, Canada; karima.afif@fsaa.ulaval.ca (K.A.); maurice.doyon@eac.ulaval.ca (M.D.)

**Keywords:** remote indigenous communities, perishable food supply chain management, food access, food security, public health, conceptual framework

## Abstract

Remote Indigenous communities experience persistent inequities in access to fresh and nutritious foods due to the fragility of perishable food supply chains (PFSCs). Disruptions across procurement, transportation, storage, retail, and limited local production restrict access to perishable foods, contributing to food insecurity and diet-related health risks. This scoping literature review synthesizes evidence from 84 peer-reviewed, grey, and unpublished sources across fourteen countries to map PFSC management (PFSCM) challenges affecting food access in remote Indigenous communities worldwide and to synthesize reported practices implemented to address these challenges. PFSCM challenges were identified across all supply chain levels, and five categories of reported practices emerged: PFSC redesign strategies, forecasting and decision-support models, technological innovations, collaboration and coordination mechanisms, and targeted investments. These findings informed the development of a multi-scalar conceptual framework comprising seven interconnected PFSCM clusters that organize how reported practices are associated with multiple food access dimensions, including quantity, affordability, quality, safety, variety, and cultural acceptability. This review contributes an integrative, system-oriented synthesis of PFSCM research and provides a conceptual basis to support future scholarly inquiry, comparative inquiry, and policy-relevant discussion of food access and health equity in remote Indigenous communities.

## 1. Introduction

Indigenous communities represent approximately 6% of the global population yet experience disproportionately high rates of food insecurity [1,2,3,4,5,6]. This issue is particularly acute in remote regions, where high prices, limited variety, and poor quality of perishable foods hinder access to healthy diets [7,8,9]. For instance, food insecurity affects up to 92% of tribes in the Klamath River Basin [10] and nearly 70% of Inuit households in Nunavut, Canada [11,12]. Geographic isolation and extreme weather conditions intensify these problems by disrupting food supply chains (FSCs) [9,13,14]. In northern Canada, for example, climate change has led to temperature increases three to four times higher than the global average, amplifying environmental disturbances such as wildfires, flooding, and permafrost thaw that expose vulnerabilities in FSCs [15,16,17].

Among FSCs, perishable food supply chains (PFSCs) are especially fragile due to the temperature sensitivity, limited shelf life, and spoilage risk associated with fresh foods [18,19,20]. Failures in PFSC management (PFSCM) directly restrict access to nutritious foods, with downstream impacts on diet quality and health outcomes [9,21,22,23]. Food access, a key pillar of food security, refers to the ability of individuals and households to acquire and afford adequate, nutritious, and culturally appropriate foods [24,25]. Beyond the physical availability of food, it encompasses social, cultural, and economic dimensions, ensuring that food is safe, adequate, and culturally appropriate [25]. In remote Indigenous regions, inequitable food access underpins high rates of nutrition-related diseases and diet-sensitive chronic conditions [22,26,27,28]. Strengthening PFSCM is therefore not merely a logistical goal but a public health imperative [29,30]. While numerous studies have documented the social and economic determinants of food insecurity in Indigenous contexts [31,32], fewer have examined how supply chain dynamics, including procurement, transportation, distribution, and retail, interact to shape access to perishable foods [23,33]. A deeper understanding of PFSC is necessary to design effective interventions that improve both supply reliability and food equity [34,35,36].

Addressing PFSCM challenges requires a systems-oriented approach that considers the interconnections among logistical, operational, and managerial subsystems across all PFSC levels [34,37]. Supply chain management (SCM) encompasses forecasting, procurement, production planning, warehousing, distribution, and retail coordination [38,39]. Isolated or single-level interventions often yield temporary solutions, whereas holistic, multi-level strategies enhance efficiency and resilience [35,36]. Adopting a systems perspective, this review maps how PFSCM challenges and practices are conceptualized, discussed, and organized in the literature, with a focus on food access and resilience in remote Indigenous contexts. According to the Supply Chain Council [40], SCM practices are “unique ways to configure, automate, or perform a set of processes that result in significantly better results.” Building on this definition and the Supply Chain Operations Reference (SCOR) model [40], PFSCM practices in this review are interpreted broadly as SCM solutions, interventions, or strategies reported in the literature across PFSC levels that address PFSCM challenges and enhance access to perishable foods in remote Indigenous communities.

A preliminary search revealed no current or ongoing systematic reviews or scoping reviews on the topic. Four existing reviews have concentrated on food and nutrition policies [31], socioeconomic interventions to access healthy food [32], retail food environments and their influence on diet-related health outcomes and obesity [41], and challenges associated with retail food environments, such as limited store access, poor food quality, and colonialism [23]. While these reviews documented barriers to healthy eating and social and cultural interventions to increase intake of fresh food, they overlooked the specific operational and logistical challenges of supplying perishable food to these regions. Furthermore, previous reviews have remained narrowly focused on retail-specific issues [23,32,41]. However, addressing the challenges of supplying perishable food to these communities requires a broader perspective on PFSC levels that encompasses procurement and supply, transportation, distribution, retail, and household consumption [34]. Previous reviews also overlooked the PFSCM practices implemented to tackle these challenges [23,32]. Additionally, these studies primarily focus on Indigenous communities in high-income countries, such as Australia, the United States, and Canada, and exclude middle- and low-income countries where similar PFSCM challenges may also occur and where reported practices could be standardized and adopted by decision-makers [23,32].

This review addresses these gaps by conducting a scoping literature review of PFSCM in remote Indigenous communities worldwide. The objectives are threefold: (i) to identify and classify the PFSCM challenges affecting food access across all supply chain levels, from procurement and transportation to retail and household consumption, (ii) to synthesize PFSCM practices reported in the literature to address these challenges, and (iii) to synthesize and organize reported PFSCM challenges and practices into an integrative conceptual framework that highlights their interdependencies across supply chain levels and food access components. Scoping review is the most appropriate method for this study, as it systematically maps existing evidence, identifies key concepts and theories, and highlights knowledge gaps [42,43]. Adopting a systems perspective, this review moves beyond descriptive accounts of remoteness or high food costs to examine how PFSCM challenges and reported practices are discussed and interconnected in the literature regarding food access. By integrating operational, socio-economic, and environmental dimensions, this study advances a multi-scalar understanding of PFSC resilience as a contextual factor shaping food access and public health in remote Indigenous communities, while providing an evidence-informed foundation to support future empirical research, policy dialogue, and context-specific inquiry.

## 2. Materials and Methods

A systematic scoping review of peer-reviewed and grey literature was conducted to synthesize knowledge on PFSCM in remote Indigenous communities worldwide. Consistent with scoping review methodology, the review included heterogeneous sources of evidence, including peer-reviewed articles, reports, and other grey literature. Non-empirical sources were used to identify recurring themes, contextual insights, and reported PFSCM practices. All included sources were treated as descriptive evidence and synthesized qualitatively without weighting by methodological rigor [42,44]. This review aimed to identify the challenges associated with supplying perishable food to these regions, the PFSCM practices reported in the literature to address them, and the gaps requiring further research. The proposed scoping review was conducted using the JBI methodology for scoping reviews [45,46]. The review structure and reporting adhered to the Preferred Reporting Items for Systematic Reviews and Meta-Analyses extension for Scoping Reviews (PRISMA-ScR) guidelines [42,47]. The completed PRISMA-ScR checklist is provided in the Supplementary Materials, Table S1.

The research questions were developed using the Population/Phenomenon, Concept, and Context (PCC) framework [48]. The phenomenon under investigation is PFSCM, a concept related to perishable food access, and the context involves remote Indigenous communities across various global regions (see Supplementary Materials, Table S2). The research questions are as follows:

RQ1. What are the PFSCM challenges that affect food access components in remote Indigenous communities across global regions, and at which supply chain levels do these challenges occur?
RQ2. What PFSCM practices have been implemented to address these challenges?
RQ3. How can reported PFSCM challenges and practices be synthesized and organized into an integrative conceptual framework?

### 2.1. Eligibility Criteria

The eligibility criteria ensured that the sources included in the scoping review were relevant to the research questions (see Supplementary Materials, Table S3). The search was limited to English-language sources published over the past thirty years (1996–2024). The starting year aligns with a seminal FAO work defining food access as a core pillar of food security, emphasizing the ability of individuals and households to obtain sufficient, safe, and nutritious food [24]. This scoping review encompasses both published and unpublished articles across various study designs, including qualitative, quantitative, and mixed methods studies. Reports, commentaries, and grey literature are included. Text and opinion papers are eligible for inclusion. Study protocols are excluded because they lack empirical evidence to address the research questions. This scoping review aims to identify the PFSCM challenges and practices for addressing them in remote Indigenous areas worldwide. Therefore, high-, middle-, and low-income countries with recognized remote Indigenous peoples, those who are the original or earliest known inhabitants of those regions, were included [1]. Sources focusing on perishable foods include both general categories (all perishable foods) and specific categories, such as fruits and vegetables. Non-food items, such as cigarettes and alcohol, are excluded. Studies focusing on traditional foods and harvesting (agriculture, fisheries) that are not sold in stores and do not involve monetary exchange are excluded. Studies assessing health, diet, and food security that are not explicitly linked to PFSCM are excluded. Public health initiatives that do not explicitly consider PFSC interventions are excluded.

### 2.2. Search Strategy

A three-stage search strategy was used. First, a limited initial search was conducted in two major online databases, MEDLINE (Ovid) and Web of Science, to find relevant articles. Second, the keywords from the titles and abstracts of these articles, along with the index terms, were used to perform a second search across all selected databases. Third, the reference lists of included sources were examined to find additional relevant research. The databases included in the search are IEEE, MEDLINE (Ovid), ABI/Inform Global (ProQuest), CAB Abstracts, and Web of Science. Sources of unpublished studies and grey literature were identified via the Grey Source Index, Web of Conferences, government publications focusing on countries with remote Indigenous communities, OpenDOAR, OpenAIRE, and SSRN. A systematic search was conducted using a combination of keywords covering all three concepts in this scoping review: remote Indigenous communities, PFSCM, and perishable food access (see Supplementary Materials, Table S4). The search strategy was tailored for each database and source. A pilot search was conducted to assess the suitability of the selected electronic databases and keywords (see Supplementary Materials, Table S5).

### 2.3. Screening

**Figure 1:**
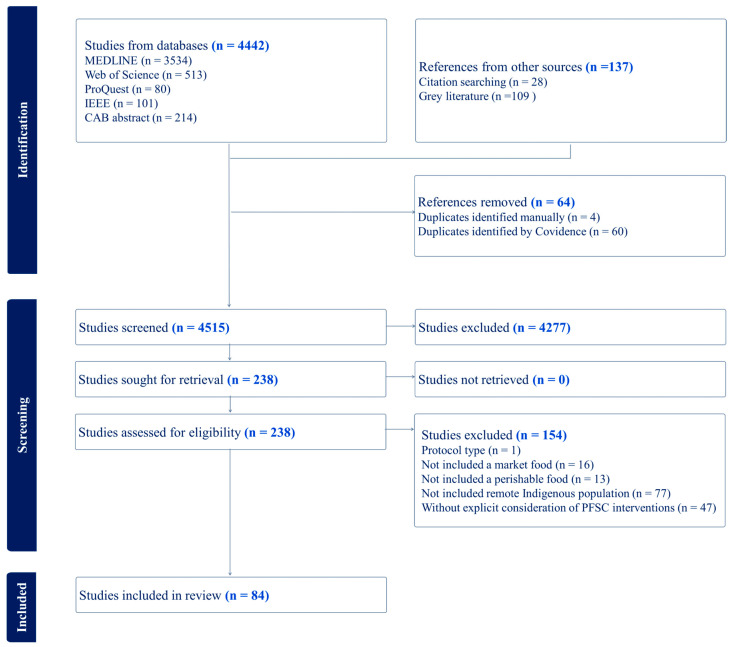
PRISMA flow chart of included and excluded articles.

The search strategy yielded 4579 potentially relevant records for screening. The final search results were uploaded to Covidence for screening. After removing duplicates (60 detected by Covidence and four by the reviewers), the titles and abstracts of all articles were screened by two independent reviewers to assess their compliance with the inclusion and exclusion criteria for the review, resolving any discrepancies through discussion. Page titles, descriptions, and initial screens from website searches were evaluated for relevance. The web addresses (URLs) of relevant records were saved in an Excel spreadsheet for full-text review. Records meeting the screening criteria (*n* = 238) moved on to the second screening, where two independent reviewers examined the full texts of selected citations in detail according to the inclusion criteria. Reasons for excluding sources that do not meet the inclusion criteria were recorded and reported in the scoping review. A total of 84 records were selected for data extraction and analysis. A PRISMA flow diagram illustrates the search results and the process of resource inclusion [49] (Figure 1).

### 2.4. Data Extraction

For the sources that met our inclusion criteria, data were extracted by two independent reviewers using a structured data extraction questionnaire (see Supplementary Materials, Table S6). The full search strategy, eligibility criteria, data extraction questionnaire, and the PRISMA-ScR checklist are provided in the Supplementary Materials and are also available via the repository DOI to support transparency and reproducibility. In accordance with the specified objectives of the review, only data pertinent to the PFSCM and food access were extracted. In addition to the study details (e.g., authors, year of publication, country, geography, population, study design, and methodology), the review includes the study purpose, specifics on the food type, supply chain levels, food access components, main findings, PFSCM challenges, identified practices, limitations of the studies, and future research recommendations aligned with the objectives of this review. The extracted data were used to summarize PFSCM challenges across different levels of the supply chain, along with the reported practices implemented to address them.

## 3. Results

### 3.1. Descriptive Characteristics of Included Sources

**Figure 2:**
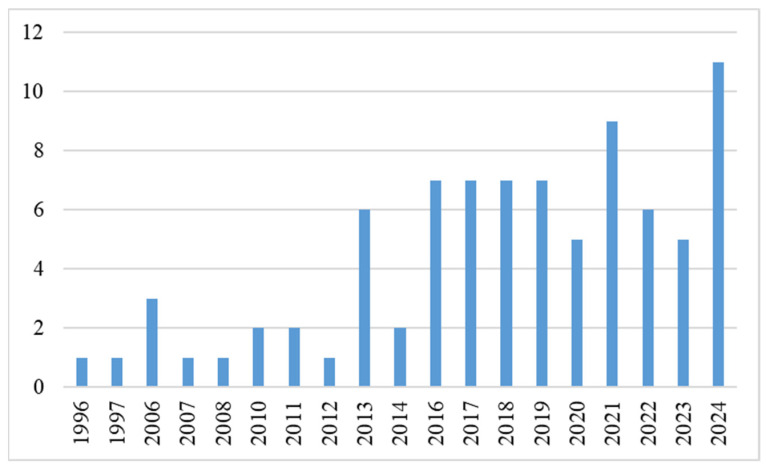
Included sources by year of publication.

Figure 2 illustrates the distribution of sources by publication year, highlighting a significant rise in PFSCM research in recent years, with a peak in 2024 (13%). This growth mirrors increased academic and policy interest in food logistics and access for remote Indigenous communities, driven by advances in cold chain and storage technology [50,51] and disruptions during the COVID-19 pandemic that revealed vulnerabilities in perishable food distribution [52].

**Figure 3:**
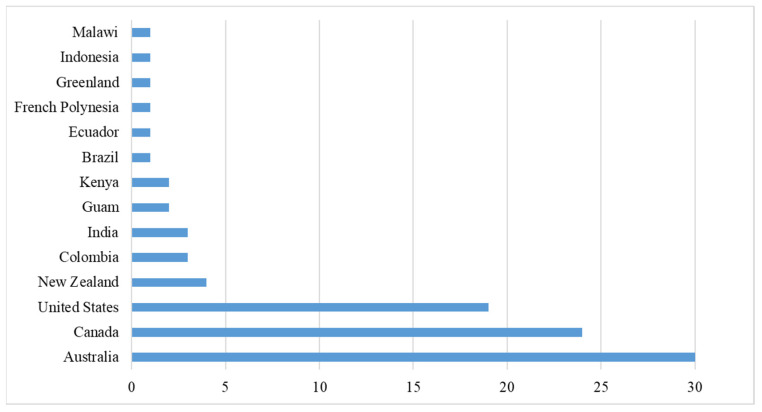
Included sources by country.

Most studies included are conducted in high-income countries, with Australia (36%), Canada (29%), and the United States (23%) being the most prominent, while there is limited representation from middle- and low-income settings (Figure 3). Other studies were carried out in Brazil (*n* = 1), Colombia (*n* = 3), Ecuador (*n* = 1), French Polynesia (*n* = 1), Greenland (*n* = 1), Guam (*n* = 2), India (*n* = 3), Indonesia (*n* = 1), Kenya (*n* = 2), Malawi (*n* = 1), and New Zealand (*n* = 4). This geographic distribution highlights the focus of PFSCM research on developed economies, despite the importance of these issues to underserved regions.

**Figure 4:**
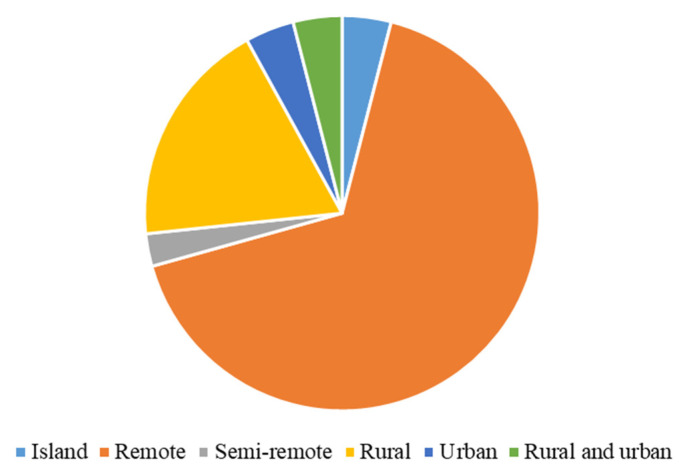
Included sources by geography focus.

Most included sources focus on remote (*n* = 50), rural (*n* = 14), semi-remote (*n* = 2), and island (*n* = 3) settings (Figure 4). Few sources address urban (*n* = 3) or mixed urban–rural (*n* = 3) areas. The sources represent a diverse range of Indigenous communities, including Aboriginal and Torres Strait Islander communities (*n* = 30), First Nations, Métis, and Inuit communities (*n* = 24), and American Indian, Alaska Native, and Tribal communities (*n* = 13). They also include Māori, Navajo Nation, and Waimānalo communities, as well as other Indigenous groups from regions such as Bora-Bora, Caliata, and the Sibundoy Valley.

**Figure 5:**
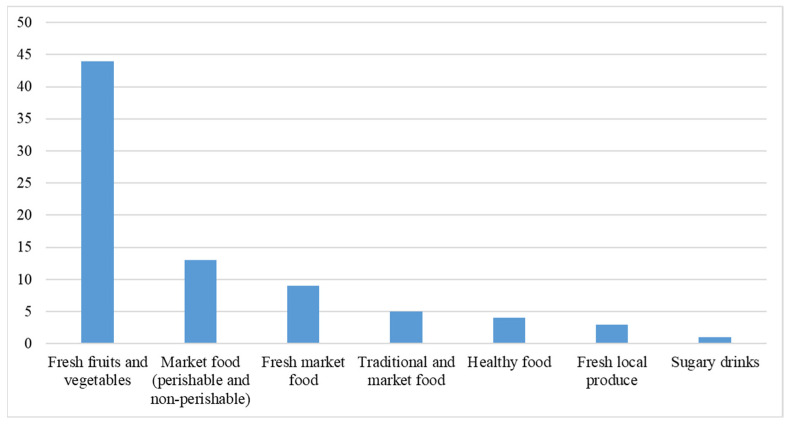
Included sources by food type.

Over half of the sources (53%) focused on fresh fruits and vegetables (Figure 5). A smaller proportion, comprising 13%, addressed both perishable and non-perishable market foods. Meanwhile, other sources focused on various fresh-market foods, including milk and meat, which accounted for 11%. Moreover, some sources emphasized healthy foods, such as whole grains, reduced-fat dairy, and low-calorie beverages (*n* = 4), while others highlighted unhealthy foods, including sugary drinks (*n* = 1), fresh local produce (*n* = 3), and traditional foods in conjunction with market foods (*n* = 5).

**Figure 6:**
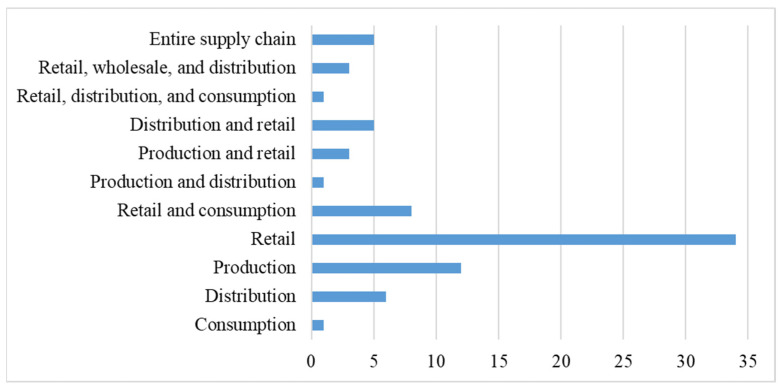
Included sources by the PFSC levels.

PFSCM analysis was most common at the retail level (*n* = 34), followed by production (*n* = 12) and distribution (*n* = 6) (Figure 6). A smaller number of sources were examined across wholesale, retail, and distribution (*n* = 3). Only a few studies adopted an end-to-end supply chain perspective (*n* = 5).

**Figure 7:**
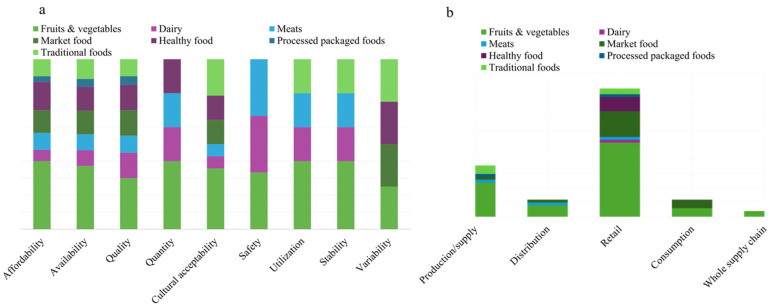
Included sources by food access dimensions, PFSC levels, and food type: (**a**) food access components examined across different food types; (**b**) PFSC levels and food types emphasized in the literature.

Food affordability (*n* = 13) and availability (*n* = 13), often examined together (*n* = 17), dominated the literature (Figure 7a). Some sources included other food access components, such as studies on quality, affordability, and availability (*n* = 22), and studies on cultural acceptability, affordability, and availability (*n* = 5). A few sources adopted a broader approach, incorporating utilization, stability, quantity, and promotion (*n* = 2).

Figure 7a shows the components of food access studied across different food types. Availability (*n* = 16) and affordability (*n* = 12) of fruits and vegetables are the most commonly examined dimensions among the sources included. Both availability and affordability have also been studied for both perishable and non-perishable market foods (*n* = 10) and healthy foods (*n* = 11). In contrast, food quality, safety, and variability related to dairy and meats are less frequently addressed (*n* = 3). We synthesized the existing literature to identify the main food categories and PFSC levels emphasized by the included sources (Figure 7b). Challenges at the retail (*n* = 26) and production (*n* = 12) levels concerning access to fruits and vegetables were frequently discussed. Similarly, retail-level challenges (*n* = 9) were often addressed concerning market foods, including both perishable and non-perishable items. Dairy and meat received comparatively less attention (*n* = 4), and research covering entire supply chains across all food types remains limited.

### 3.2. PFSCM Challenges in Remote Indigenous Communities

**Figure 8:**
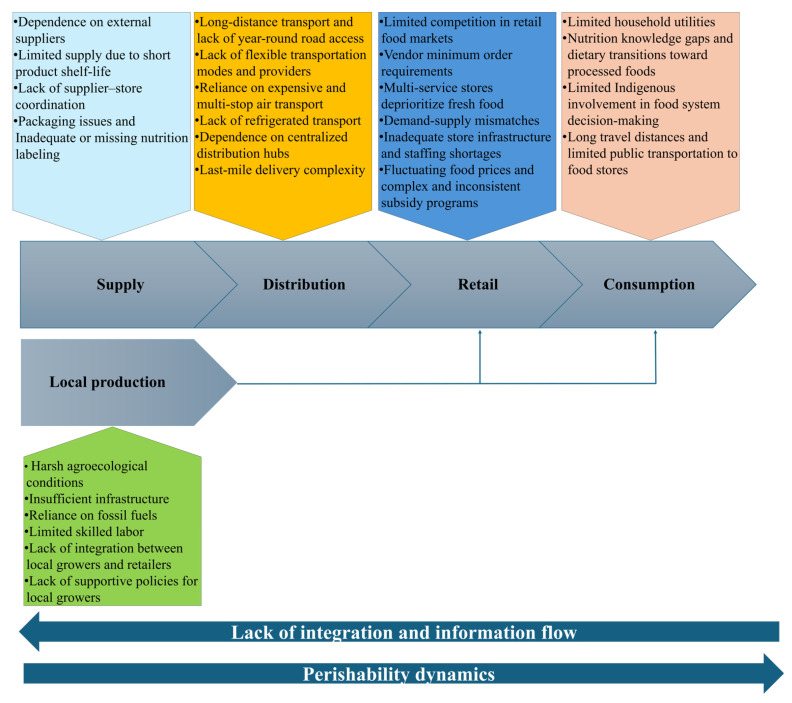
PFSCM challenges in remote Indigenous communities across interconnected supply chain levels. Arrows indicate supply chain flows, with increasing perishability toward the consumption level and lack of downstream-to-upstream integration and information flow.

Figure 8 summarizes the main PFSCM challenges in remote Indigenous communities globally (RQ1). These challenges span five interconnected levels: external supply, local production, distribution, retail, and consumption, shaping food access in these contexts.

#### 3.2.1. Local Production and External Supply Challenges

PFSC typically begins with external suppliers due to the limited local production capacity within remote Indigenous regions across Canada [53,54,55,56,57,58], the United States [59,60,61], Australia [9,62], Indonesia [63], India [64], Kenya [65,66], Brazil [67], and Colombia [50]. Consequently, these communities are heavily dependent on external suppliers, which raises their vulnerability to supply chain disruptions [68]. For instance, in Western Australia, the lack of local production necessitates importing food from the eastern regions of the country, leading to higher prices and limited variety [9].

Remote Indigenous communities face interconnected challenges that threaten the sustainability of local food production, including agricultural and gardening initiatives [54,55,69]. Severe agroecological conditions, such as poor soil quality, climate variability, harsh weather, and water scarcity, hinder consistent farming [50,56,57]. In Australia, remote communities often lack the essential infrastructure for reliable water access [62]. Moreover, access to arable land remains a significant obstacle. For Indigenous communities across Brazil, land scarcity increasingly limits gardening initiatives [67]. Extreme weather also limited growing periods in regions such as Canada, the United States, Australia, and Colombia (e.g., [13,56,57]). As a result, the availability and affordability of fresh produce, such as fruits and vegetables, are highly inconsistent throughout the year in these regions [70,71]. Innovative methods are emerging to address these challenges: in the United States, the MALAMA program combines aquaponics with Native Hawaiian cultural practices to increase year-round growing capacity [61]. Researchers in a rural Colombian community utilized a machine learning-based digital map to assess the agroecological potential, thereby improving access to locally grown food [50].

Local growers face knowledge gaps in seed and soil quality, crop selection, and the technologies [50,54,55]. A lack of skilled labor also hinders local food production efforts. For example, in West Papua, limited technical expertise, from cultivation to harvest, has hindered the sustainable local food systems [63]. Even when food is successfully grown, inadequate post-harvest infrastructure, such as cold storage, refrigeration, and processing facilities, leads to significant spoilage [64,66,68,72]. Poor handling practices in low- and middle-income Indigenous regions, such as washing vegetables with contaminated water or exposing them to direct sunlight, further compromise food quality, reduce nutrient content, and jeopardize safety (e.g., [72]). Moreover, the absence of transportation networks and centralized local markets limits the market for locally produced food [66,68,73]. Retailers often hesitate to buy from local producers due to insurance restrictions and strict quality, safety, and pricing standards [19].

Remote food stores rely on a limited number of suppliers, resulting in reduced variety and higher costs for nutritious foods, as observed in the Canadian Arctic and the Navajo Nation [28,74,75]. A lack of coordination between suppliers and community stores further restricts access to fresh, nutritious food. In remote areas, ensuring the consistent availability of essential nutritional products requires coordinated logistical planning between store managers and external suppliers (e.g., [28]). Additionally, in many remote Pacific nations, imported foods often lack clear nutrition labels [70]. In New Zealand, affordable food items consumed by low-income Indigenous populations often lack accessible, meaningful nutritional information [76]. Furthermore, supplying remote communities requires considerable packaging, ranging from disposable wooden or cardboard boxes to reusable plastic crates. In areas like Nunavik, the absence of recycling infrastructure renders life-cycle assessment models impractical, thereby impeding efforts to evaluate the long-term environmental impacts of these materials [56].

#### 3.2.2. Distribution-Level Challenges

Remote Indigenous communities across Australia [9,21,77,78], Canada [5,79,80,81], the United States [82], French Polynesia [73], and India [83] face significant challenges in food distribution. Table 1 presents a context-specific synthesis of distribution-level challenges and their corresponding impacts on the key components of food access.

Challenges associated with long-distance distribution are frequently cited in the included sources, affecting the availability, cost, variety, and quality of fresh food (e.g., [9,21,58,77,84,88]). As remoteness increases, food quality declines and prices rise (e.g., [5,9,81]). Insufficient distribution infrastructure has led to food insecurity rates reaching 46.4% in some First Nations communities across British Columbia, Alberta, Manitoba, and Ontario [81].

The lack of year-round road access remains one of the most critical challenges to reliable food distribution in remote Indigenous regions (e.g., [78,81]). In northern Canadian communities, this limitation substantially increases transportation and distribution costs, thereby increasing food prices and reducing the consistency of fresh food availability [5,80,81,85]. In subarctic Ontario, the construction of an all-season road has been proposed to enhance both food availability and affordability [58]. Comparable logistical bottlenecks, particularly the absence of all-weather road networks, similarly constrain access to perishable foods in remote communities across Australia and Greenland [28,78].

Harsh weather further exacerbates distribution challenges by causing delays, affecting food quality [28,79,88]. Extreme temperatures can prolong distribution times by up to tenfold, as observed in the Canadian Northern Territory and Western Australia (e.g., [9,13]). Similarly, unpredictable weather in Greenland disrupts food distribution (e.g., [28]). Severe weather often causes lost or delayed orders, resulting in perishable foods arriving in poor condition and being sold at reduced prices or not at all. In remote Inuit communities, fresh foods freeze during distribution and deteriorate in quality throughout the 10-month Arctic winter [79]. Storekeepers often increase prices to offset spoilage-related losses, making fresh food even less affordable for Indigenous consumers [86].

Moreover, food distribution to remote communities relies on limited transport modes. The modes of air, sea, rail, and road transportation impact fresh food pricing differently [33,87]. The food supply to remote communities in Western Australia relies on long-haul road or rail transport [9]. Remote Inuit communities primarily depend on two modes of transportation: air freight and barge. Due to weather conditions and disruptions caused by sea ice, fresh foods with short shelf lives, such as fruits and vegetables, are restocked weekly via costly air freight [79]. As a result, market food prices in Arctic Indigenous communities are two to three times higher than in southern regions [75,79]. Increasing competition among airlines and suppliers could help lower freight costs, making it more affordable to transport perishable food to these communities [75]. Similarly, communities in the Yukon-Kuskokwim Region of rural Alaska are off the road and depend on small, expensive aircraft for food deliveries. These flights are often delayed by severe weather, which decreases the quality of fresh food in these regions [86].

Centralized distribution systems also impact the food distribution to these communities. In rural and remote Queensland, long distances from centralized hubs, such as Brisbane, contribute to high food prices [21]. Extended distribution distances, combined with adverse weather conditions and infrastructural limitations, result in fragile distribution systems (e.g., [13,68,89]). Additionally, cold chain systems during distribution are often lacking or unaffordable in these areas (e.g., [9,68]). In northern Canada, the absence of cold chain systems during air transport further compromises the quality of fruits, vegetables, and dairy products [87]. Multi-stop air deliveries to remote Alaskan communities often result in fruits and vegetables arriving wilted, moldy, or spoiled shortly after delivery [75,86]. In Aboriginal and Torres Strait Islander communities, the costly long-haul refrigerated transport limits the feasibility of long-term improvements in fresh food access [77], causing fresh food prices in these remote regions to rise by as much as 30% when compared to urban areas [74]. Fuel costs further amplify these challenges. Fuel expenses directly affect food prices and, indirectly, food availability in remote communities [84,87]. Even communities with road access in northern Manitoba experience significantly higher food prices due to soaring fuel costs [5]. Paz-Orozco et al. (2022) [51] demonstrated that optimizing food distribution routes can reduce fuel-related transport costs by nearly 29% in remote areas in Colombia.

#### 3.2.3. Retail-Level Challenges

Limited competition within retail markets constitutes a significant barrier to food accessibility in remote Indigenous communities, limiting the availability and diversity of fresh produce (e.g., [5,90]). For example, Wapekeka, a remote First Nations community in Ontario, has only one grocery store [91]. In over fifty percent of northern Canadian communities, the Northwest Company serves as the sole grocery provider [90]. This retail monopoly often lasts for prolonged periods, up to 9 or 10 months, particularly when seasonal winter roads become inaccessible [91]. Similar issues have been documented in remote regions of Western Australia, where limited market competition and reliance on small, independent stores result in diminished food access [9,74].

Store type impacts access to fresh food in remote areas. Large retailers offer a wider variety of products and lower prices. At the same time, small stores, which are crucial in rural areas, often sell lower-quality items at higher prices and less fresh produce [71,92,93,94]. For instance, in subarctic Ontario, Canada, there is only one store that provides various services, including banking, but offers limited fresh food options [58]. Gitxaala, a remote First Nations community in British Columbia, has access only to small convenience stores with a limited food variety, such as milk bags and canned goods [91]. In contrast, grocery stores in the Navajo Nation offer a broader array of fresh food, including fruits and vegetables, making consumers 520% more likely to purchase them compared to convenience stores [95]. Community-owned supermarkets have proven effective in improving food availability, quality, and pricing [58,92]. Stores with strong community ties are 120% more likely to sell fresh produce than convenience stores [95]. In low- and middle-income countries, access to fresh food often depends on informal markets. For instance, remote communities in India rely on small kiosks that provide high-quality, locally produced foods [64]. Similarly, in remote regions of Guam, small outlets supply more fresh fruits and vegetables than other types of stores [94].

Furthermore, irregular deliveries and minimum-order requirements imposed by vendors pose significant challenges for remote stores [19]. For instance, in remote Navajo communities, fresh food is distributed through several intermediate distributors, each charging delivery fees and minimum order quantities [19]. Corporate stores in the Yukon-Kuskokwim region of Alaska are required to purchase from specific vendors, which limits their ability to diversify food selections or adapt quickly to consumer demands [86]. Moreover, delivery delays further reduce the quality of fresh produce, resulting in substantial food waste. In isolated areas of Greenland, perishable foods spoiled in 2016 due to delayed deliveries [28]. Infrequent food deliveries contribute to high prices, poor quality, and limited food variety [9,75]. Retailers within the Navajo Nation collaborate with local grocery stores to place bulk orders from larger distributors, thereby enhancing their purchasing power and decreasing per-unit costs [19]. Food distributors in Northern Greenland are considering adjustments to delivery schedules and supply coordination strategies to address these challenges [28].

Inadequate infrastructure contributes to increased operational challenges for remote retail stores, which, in turn, reduces food quality and availability in these communities [9,79,87,96,97]. A limited or unreliable electricity supply disrupts refrigeration and food storage, exacerbating the challenge of maintaining fresh food stocks (e.g., [87,96]). Some stores depend on backup generators, but these are not always sustainable or cost-effective (e.g., [87]). Infrastructure-related issues, such as malfunctioning refrigerators, are challenging to resolve, forcing store managers to make difficult decisions about which foods to stock or remove, decisions that, in turn, further affect the availability and quality of fresh food [79]. These issues are further exacerbated by frequent store closures, irregular delivery hours, limited storage capacity, and staffing shortages [80,86,97].

Fluctuating prices affect access to perishable food in these areas [71,98,99]. For instance, stores in remote Australian regions that supply seasonal, fresh produce from large national wholesalers face unpredictable prices and struggle to compete with supermarket chains that sell non-perishable, packaged food [98]. However, ensuring access to fresh food in these communities involves more than simply supplying fresh food [9]. Demand-supply mismatches pose significant challenges. Inaccurate demand forecasting often leads to overstocking or shortages, resulting in waste, spoilage, and reduced affordability [78,90]. Seasonal changes that decrease consumer demand increase uncertainty within PFSCM [71]. The fluctuating price elasticity in these communities further complicates the demand for fresh food, making it challenging for store managers to develop effective pricing strategies [87]. A comprehensive study conducted in remote Indigenous communities in Australia found that a 20% discount on fresh produce had a minimal effect on purchasing behavior [28,99]. Store managers in Australia and Greenland noted that pricing strategies alone do not significantly boost sales of fresh food [28,100,101]. Pricing strategies should account for the size and duration of the discount, as well as the promotional methods employed [101].

To improve food pricing policies, involve key PFSC stakeholders in planning and monitoring promotional strategies [100]. Machine learning and deep learning enhance forecasting accuracy, and collaborative forecasting with suppliers, retailers, and consumers matches supply and demand [102]. Information and Communication Technologies (ICTs) facilitate improved collection, storage, and sharing of information, sustain demand forecasting for fresh food, and effectively adjust food prices [103]. Most (81%) of the stores in remote Western Australian Indigenous communities already utilize electronic point-of-sale systems to manage their financial and retail operations efficiently [9]. Enhancing these systems optimizes retail management practices and enhances initiatives such as revenue management and store intervention assessments [102]. Table 2 summarizes challenges associated with the retail level across regions and their impacts on food access components.

#### 3.2.4. Consumption-Level Challenges

Long travel distances to grocery stores and limited public transportation significantly hinder food access for Indigenous communities in remote regions, including those in Canada and Australia (e.g., [84,104]). Residents in rural areas often travel considerable distances to purchase groceries, incurring additional costs related to time, fuel, and vehicle maintenance [84,105]. In Canada, subsidized food programs, such as the Food Mail Program, aim to reduce logistical barriers; however, implementation issues have limited their overall success [80]. Locating grocery stores near public transportation hubs enhances accessibility for Indigenous individuals without private vehicles, as demonstrated by the West End Farmers Market in Atlanta [59]. Similarly, grocery store location models that account for population density, such as those used in Western Australia, have been employed to enhance access to fresh produce [9]. Initiatives like mobile markets in U.S. Indigenous communities, such as the Osage Nation, have proven effective by increasing direct-to-consumer access [82].

Broader socioeconomic factors, including income, cultural practices, and household resources, also impact fresh food demand in these communities [87]. Unreliable electricity and water supplies, as well as insufficient food storage, significantly hinder the purchasing power of households in remote Aboriginal and Torres Strait Islander communities [87]. Food storage options varied among households: some families purchased in bulk and utilized walk-in coolers or chest freezers, whereas others depended on small refrigerators. Consequently, many residents relied on local food outlets as their improvised “storeroom” (e.g., [74,87]). The shift from conventional diets to commercially available foods has also altered purchasing behaviors within these communities (e.g., [84,99]). In Nunavut, Inuit communities are increasingly reliant on prepared foods (e.g., [79,90]). Retail management practices, such as placing fresh produce near the checkout, utilizing culturally relevant promotional materials, and training staff to recommend healthier options, have been implemented to mitigate this issue (e.g., [98,106]). In Western Australia, some stores use alternative purchasing systems, such as “book-down” arrangements, in which customers deposit funds in advance to secure future food purchases. However, concerns about repayment and financial instability persist, limiting the broader adoption of such practices [9]. These practices have demonstrated limited success unless paired with nutrition education and culturally relevant marketing [99,107].

Many remote communities have limited participation in food system decisions, resulting in policies and PFSC practices that do not align with local needs [68,108]. Decision-making frameworks should foster collaboration with Indigenous store owners, nutritionists, and community stakeholders to address this gap and ensure culturally appropriate and effective practices [100,101]. In remote Indigenous communities in Western Australia, only 22% of store managers have reported collaborating with a local nutritionist [9]. The store ownership and management structure, whether through Aboriginal corporations, private ownership, store groups, or independent operators, has a significant impact on store operations, purchasing capacity, and market power [19,87]. Storekeepers in the Yukon-Kuskokwim Region of Alaska reported the importance of traditional food systems and subsistence practices in preserving cultural identity and promoting nutritional well-being [86].

### 3.3. PFSCM Practices in Remote Indigenous Communities

**Figure 9:**
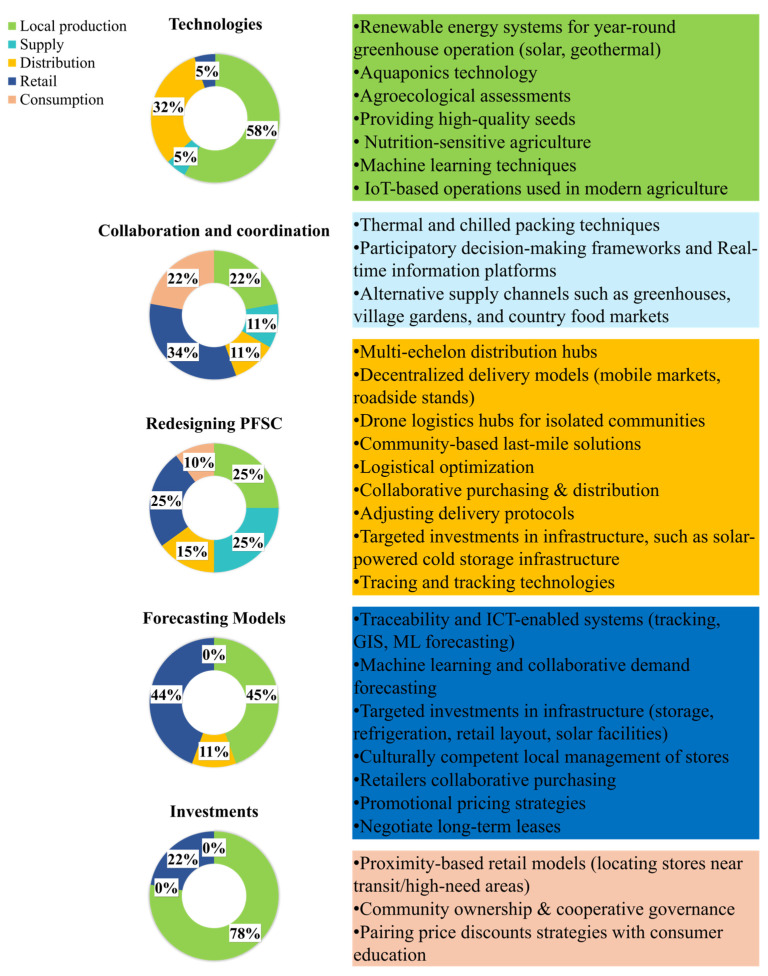
Reported practices implemented to address the PFSCM challenges in remote Indigenous communities.

A thematic analysis was conducted to categorize the practices implemented to address PFSCM challenges in remote Indigenous communities worldwide. These practices were classified into five categories, including redesigning PFSC, forecasting models, technologies, collaboration and coordination, and investments (RQ2) (Figure 9). Challenges can be addressed individually or with multiple practice categories. Each category is represented in a pie chart illustrating the distribution of these practices across various PFSC levels. For example, in the technology-related pie chart, 58% of technological practices are enacted at the production level, 5% at the supply level, 32% at the distribution level, and 5% at the retail level.

#### 3.3.1. PFSC Redesigning Strategies

These strategies reduce reliance on external suppliers through community-driven initiatives, such as greenhouses, gardens, and village farms, which improve food availability, affordability, and cultural acceptability [52,55,58,60]. Community greenhouses in Nunavik, Nunavut, and subarctic Ontario produce fresh, affordable food while reconnecting communities with traditional farming practices (e.g., [55,58]). In Saskatchewan, grassroots food preservation methods extend access to nutritious foods throughout winter [52]. In Alaska, village gardens support partial food self-sufficiency in remote areas [60]. However, these initiatives often require much energy, relying on diesel-powered systems that increase costs and reduce sustainability [54]. Renewable energy options, including solar thermal panels and geothermal heating, are increasingly being utilized to address these challenges and support year-round greenhouse operations [54,56,57].

Decentralized and flexible distribution systems can improve PFSC effectiveness in remote areas (e.g., [109,110]). A multi-echelon hub distribution structure is particularly relevant in contexts such as Northern Canada, where multimodal transportation is typical [14]. These hubs can consolidate or redistribute food flows, thereby improving transport economies of scale (e.g., [109]). Only a few studies have examined hub-based delivery strategies for addressing this issue [109,110,111]. Frank et al. (2021) [110] proposed a framework for locating multimodal mobility hubs based on transport modes, distance, and usage patterns. Pan et al. (2020) [111] introduced algorithms for strategically placed drone logistics hubs, offering a potential solution for reaching highly isolated communities. Direct-to-customer models, such as roadside stands and mobile markets, are more effective than centralized retail stores in rural areas [59,73,82,112]. Similarly, proximity-based retail strategies that locate grocery stores and food markets near public transportation hubs or high-need areas have improved food accessibility [9,59]. Table 3 summarizes these context-sensitive strategies and their effects on food access.

#### 3.3.2. Forecasting and Optimization Models

Recent innovations in forecasting, particularly machine learning and collaborative planning, have significantly improved the accuracy of perishable food demand prediction in remote Indigenous PFSC [90,102]. Additionally, the literature highlights that improving the efficiency of distribution systems and strategic location-allocation modelling can enhance accessibility [14,113]. Accessibility can be modeled mathematically. For example, Frank et al. (2021) [110] proposed a dual-objective model that maximizes travel reachability while minimizing distance to key transportation hubs, incorporating mobility infrastructure into the optimization. Lin et al. (2012) [114] showed that introducing new support facilities or reconfiguring existing ones can increase both distribution efficiency and accessibility in rural supply chains. Escribano Macías et al. (2020) [115] developed a drone delivery system that considers terrain and initial network access, thereby improving physical connectivity in remote regions.

Distribution planning in remote areas should extend beyond basic route optimization models, which typically consider only general constraints such as vehicle capacity, facility limitations, and inventory levels [14]. It should also consider contextual constraints, such as seasonal accessibility and poor infrastructure, which make choosing transportation modes a strategic decision influenced by terrain and environmental conditions (e.g., [5,80,81,85]). Optimization models that support such planning by evaluating road feasibility, network configuration, and the trade-offs between construction costs, accessibility, and service coverage (e.g., [116,117]) enable decision-makers to approach rural infrastructure development from a data-driven, location-allocation perspective (e.g., [51,113]). Davis et al. (2014) [118] addressed perishability as a critical constraint in the PFSCs by integrating time-sensitive constraints into scheduling models to ensure food safety and the regularity of deliveries. They demonstrated that increasing the number of distribution hubs is essential for maintaining food quality in remote areas. Few studies have directly investigated food distributions under perishability constraints in rural or Indigenous contexts (e.g., [109,119,120]). Optimization models within the existing literature predominantly adopt deterministic approaches (Table 4). However, stochastic data models effectively capture the inherent uncertainty and variability of such areas [14].

#### 3.3.3. Technology Approaches

Nineteen sources included in this scoping review examined technology-driven interventions to improve food access in remote areas across the PFSC. Approximately 58% focus on localized technological solutions that improve agricultural production efficiency. These solutions include renewable energy technologies, such as solar thermal panels and photovoltaic systems, which enhance energy independence in remote areas [56,121,122]. Innovative farming systems, such as aquaponics and the integration of machine learning into cultivation and yield management, further help reduce reliance on external suppliers while improving food quality, affordability, and year-round availability (e.g., [50,61]).

Solar-powered cold storage, advanced thermal and chilled packaging, and wireless sensors for real-time condition monitoring ensure the integrity of perishable food during long-distance transportation [54,56,98]. Similarly, traceability systems and tracking technologies enhance transparency, facilitate compliance with food safety regulations, and foster consumer trust [84]. ICTs enable the collection, sharing, and analysis of accurate data, supporting timely decision-making and adaptive management among PFSC actors [102,103]. Machine learning models further improve demand forecasting by addressing mismatches between supply and demand in dynamic and unpredictable environments [90,102]. Additionally, GIS-based spatial analysis aids logistics planning by visualizing terrain constraints, enabling the development of proactive solutions to distribution challenges [51].

#### 3.3.4. Collaboration and Coordination Strategies

At the production level, nutrition-sensitive agriculture and culturally informed agrotechnology strategies strengthen the alignment between local food systems and community needs [64,66,67]. At the consumer level, price incentives, in conjunction with educational campaigns [106], and the involvement of culturally competent local managers [19,56,66,84,90] reinforce this alignment with cultural expectations. Coordination across the supply chain further supports this process through supplier–community store partnerships [28,68], collaborative purchasing mechanisms [19], and adaptive delivery protocols [28]. Finally, participatory decision-making frameworks involving suppliers, retailers, and consumers [101,102,108] facilitate the co-creation of context-specific and practically viable solutions.

#### 3.3.5. Investments

The sustainable transformation of PFSC within remote Indigenous communities depends on targeted investments across various levels of the supply chain. Tracking systems enhance food quality and safety [53,56]. Implementing solar-powered cold storage facilities enhances food quality and increases its availability [121,122]. Investing in local production, mainly with high-quality seeds, can significantly enhance the quality and diversity of fresh produce [50,72]. At the retail level, infrastructure improvements (e.g., refrigeration, storage, and layout design) ensure consumers have access to healthy options [86,95]. Policy-driven initiatives, such as the Stores Licensing Scheme in Australia, demonstrate how infrastructure standards and reliable power can systematically enhance retail performance in underserved areas [123]. Addressing financial issues, such as lease negotiations and electricity subsidies, can also lower operational costs at the retail level and support long-term sustainability [87,96].

## 4. Discussion

This scoping review synthesizes the challenges and practices of PFSCM in remote Indigenous communities, drawing on 84 sources from 14 countries. A central insight is that PFSCM stakeholders face persistent challenges due to limited integration and poor connectivity along the supply chain [19,28,75,78,90,101]. Information flow from consumers rarely reaches upstream actors, and weak retailer-community linkages prevent supply from aligning with local cultural and nutritional needs [68,101]. Dependence on imported foods exacerbates these issues, often resulting in products that are culturally inappropriate or nutritionally inadequate [70,76]. Barriers to food access in remote Indigenous communities extend beyond geographic isolation, including unreliable infrastructure, limited access to cold storage and electricity, limited nutritional awareness, and insufficient community involvement in food governance [33].

Traditional measures of food access often focus narrowly on retail availability and affordability, overlooking the broader sociotechnical context through which food is produced, distributed, and exchanged [59,74,84]. While community-based food production initiatives, such as greenhouses and local harvesting activities, are discussed as mechanisms that support food availability and cultural relevance, local growers lack consistent market channels, and retailers remain reluctant to source from small-scale Indigenous producers [19,58,66]. Beyond formal retail systems, a limited but growing body of literature points to the potential role of Alternative Food Networks (AFNs), including community-supported agriculture, food hubs, cooperatives, farmers markets (both in-person and online), and traditional food-sharing networks, as alternative or complementary supply channels that connect producers and consumers in remote contexts (e.g., [34,124,125]).

The review also highlights how inefficient flows of funding and information can constrain food access initiatives. For example, despite substantial subsidies through Nutrition North Canada (NNC), limited community awareness and a lack of transparency about program operations have constrained its impact on food affordability [126]. Similarly, the 10% GST (Goods and Services Tax) exemption on basic healthy foods in Australia had a restricted effect on enhancing food access for remote Indigenous communities [127]. These findings underscore that financial incentives alone are insufficient; sustainable PFSCM requires coordinated system design, effective communication, and active community engagement [107,126,127,128]. Policy strategies should focus on strengthening partnerships between Indigenous producers and retailers, enhancing information flows, and building community capacity [58,129].

**Figure 10:**
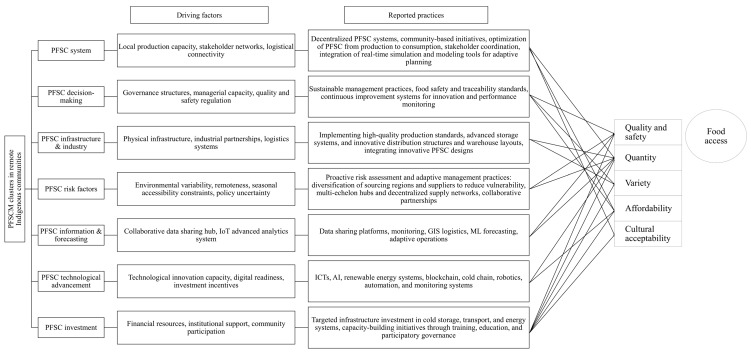
Conceptual framework for PFSCM in remote Indigenous communities, illustrating seven interconnected clusters, PFSCM practices, and food access components.

The synthesis of the reviewed literature indicates that PFSCM in remote Indigenous communities is discussed through a systems-oriented lens that emphasizes the interconnections among multiple, interdependent challenges and practices across the supply chain. The proposed conceptual framework (Figure 10) integrates and organizes how PFSCM challenges and practices are represented in the literature. The framework comprises seven interrelated thematic clusters: (1) PFSC system, (2) PFSC decision-making, (3) PFSC infrastructure and industry, (4) PFSC risk factors, (5) PFSC information management, (6) PFSC technological advancement, and (7) PFSC investment and capacity building. Building on the analytical structure proposed by Kafi et al. (2025) [37], these clusters synthesize five categories of PFSCM practices reported in the literature, including PFSC redesign strategies, forecasting models, technologies, collaboration and coordination, and investments, into an integrative, system-oriented perspective. The framework highlights how these clusters are discussed in relation to key food access components, including quantity, affordability, quality, safety, variety, and cultural acceptability.

The PFSC system (Cluster 1) captures how the literature describes the network of external suppliers, local growers, distributors, retailers, and consumers. By optimizing the flow of perishable food from production to consumption and enhancing stakeholder coordination, an efficient PFSC system ensures timely delivery while reducing waste [130]. This comprehensive view highlights the connections among PFSC levels and stakeholders, supporting resource allocation and helping prevent supply chain disruptions [37]. In remote Indigenous settings, several studies discuss integrating real-time modeling and simulation as approaches for responding to uncertain demand, environmental conditions, and distribution challenges [14,131]. Decentralized PFSC configurations, such as multi-echelon hub structures, are frequently described as alternatives to highly centralized systems to address logistical fragility and distribution challenges (e.g., [109,110,118]). Similarly, the literature reports horizontal coordination among retailers, such as collective purchasing arrangements, as a strategy discussed in relation to bulk procurement and pricing dynamics for fresh produce (e.g., [19]).

Community-based initiatives such as greenhouses, village gardens, and local farms are reported as PFSC system redesign strategies that shorten the supply chain and improve food availability, affordability, and cultural relevance in these areas [52,55,58]. These strategies, aligning with Indigenous consumer demand, help the PFSC system match supply and demand, reducing stockouts, overstock, and costly adjustments [78,90]. A redesigned Indigenous PFSC system should focus on two main goals: (1) increasing hyper-local production and policies to reduce reliance on external inputs [68,100], and (2) restructuring distribution systems to allow Indigenous producers to participate in markets and exchange networks [75,132,133]. Strengthening local food production requires addressing systemic barriers, such as limited access to productive inputs, including land, infrastructure, and equipment, which hinder self-sufficiency in many remote communities [50,55,62,134]. Recognizing and leveraging traditional food-sharing practices, which serve as informal yet resilient supply chains, not only helps address food insecurity but also strengthens social bonds and enhances the well-being of Indigenous communities [133,135].

PFSC decision-making (Cluster 2) involves the strategic coordination of all supply chain practices [136]. This cluster encompasses methods such as knowledge-based modeling, predictive analytics, and decision-support systems, which aim to enhance efficiency, reduce operational costs, and strengthen PFSC resilience [137,138]. In the reviewed literature, advanced food safety measures, such as thermal and chilled packaging and wireless sensors for real-time condition monitoring, are discussed in relation to ensuring the quality of perishable foods during extended and unpredictable transportation [54,56,98]. The success and sustainability of these decision-support approaches depend on integrating them into participatory governance frameworks that promote co-learning, shared power, and capacity-building among community members and stakeholders, mobilize knowledge for local benefit, and encourage collective ownership of PFSC decision-making [102,121,139].

PFSC infrastructure and industry (Cluster 3) captures how studies conceptualize the role of physical infrastructure and industry actors in shaping PFSC operations [81,140]. Implementing high-quality production standards, advanced storage systems, and innovative distribution structures and warehouse layouts ensures the quality, safety, and accessibility of food [141,142]. The literature discusses the integration of sustainable practices and innovative PFSC designs, such as drone delivery systems, renewable energy-powered greenhouses, and resource-efficient production methods, as context-specific responses to infrastructural constraints [56,109,110,115].

PFSC risk factors (Cluster 4) encompass the uncertainties and potential disruptions that affect the efficiency and safety of the supply chain, including environmental, economic, regulatory, and logistical risks [143]. Climate change, extreme weather, and seasonal accessibility constraints further exacerbate these risks in remote Indigenous communities [13,91,92], underscoring the need for proactive risk assessment and adaptive management strategies [142]. The literature discusses a variety of risk mitigation measures, including diversifying sourcing regions, establishing transportation redundancy, and leveraging advanced forecasting technologies [14,102]. These strategies play a crucial role in minimizing vulnerabilities and maintaining a reliable food supply. Furthermore, collaborative public–private partnerships are highlighted as mechanisms for facilitating knowledge and resource exchange among diverse stakeholders [144]. These partnerships enable collaboration among government bodies, industry stakeholders, and communities to collectively address complex risk factors, thereby enhancing the ability of the supply chain to identify and mitigate hazards and ultimately enhance the resilience of PFSC in these regions [101,102,108].

PFSC information management (Cluster 5) involves collecting, exchanging, and analyzing data within the PFSC [145,146]. This cluster enhances the ability of PFSC to respond dynamically to fluctuations in demand and supply, resource limitations, and distribution-related challenges due to environmental conditions [14,87,90,131]. Studies emphasize the role of collaborative data-sharing platforms, ICTs, GIS-based logistics planning, and machine learning forecast models in supporting transparency, coordination, and adaptive decision-making under conditions of uncertainty [102,147,148,149]. Accurate forecasting and optimization models help mitigate perishability constraints, improve distribution efficiency, and allow adaptive operations amid uncertainty [14,110,118]. However, they require adjustments to remote Indigenous context constraints, such as unpredictable distribution conditions and socio-economic variability [14,131].

PFSC technological advancement (Cluster 6) reflects how the reviewed literature discusses technological innovations such as ICTs, blockchain, cold chain management, and renewable energy systems in relation to PFSC efficiency, traceability, and sustainability [56,121,150,151]. Robotics, monitoring systems, and automation technologies are described as components that support operational coordination and quality assurance within PFSC [152,153]. These technologies support proactive decision-making, resource optimization, and risk mitigation, fostering a more resilient and responsive PFSC [154].

PFSC investment and capacity building (Cluster 7) synthesizes how financial investments are discussed as cross-cutting enablers of PFSC operations across supply chain levels. Investments, such as infrastructure improvements, capacity-building initiatives, high-quality inputs for local production, and policy-driven programs, enhance food accessibility, affordability, and cultural acceptability while ensuring the long-term sustainability of PFSC in remote indigenous areas [53,95,123]. Capacity-building efforts, including community engagement, participatory decision-making, and knowledge transfer, strengthen the adaptive capabilities and resilience of PFSC in these regions [102,128].

The conceptual framework illustrates how the seven thematic clusters identified in this review are discussed in the literature in relation to key components of food access, including quantity, affordability, quality, safety, variety, and cultural acceptability. The framework organizes how PFSCM challenges and practices are commonly associated, co-articulated, and interconnected across supply chain levels within existing studies. By synthesizing reported practices alongside food access dimensions, the framework provides an integrative and theory-informed structure that supports understanding of how PFSCM is conceptualized in remote Indigenous contexts. The framework reflects recurring patterns in the literature in which technological development, information management, decision-making processes, investment, and capacity building are discussed as interrelated and mutually reinforcing thematic domains. From a policy and public health perspective, the framework situates PFSCM as a contextual factor shaping food access and health equity. It offers a descriptive, context-sensitive lens that can inform future empirical research, comparative analysis, and policy-relevant dialogue on PFSC sustainability and food access in remote Indigenous communities.

## 5. Knowledge Gaps and Pathways for Future Research

This scoping review highlights significant gaps across four key areas: (1) scope-related, (2) methodological and analytical, (3) conceptual, and (4) geographic. First, the current literature has a limited scope, mainly focusing on retail environments and excluding local grower markets and alternative food supply channels, such as online markets [9,23,34,124,125]. Second, methodological and analytical limitations restrict the scope and applicability of existing research. At the community level, understanding food access and challenges related to PFSC remains limited, especially in informal settlements [65,155]. The included studies exhibit sampling biases, often relying on participants from urban Indigenous regions or conducting online surveys [52]. This approach may exclude remote populations without reliable internet access, raising concerns about the representativeness and generalizability of the findings. Future research should adopt inclusive designs that address geographic isolation and digital divides, improving understanding of these issues. Small sample sizes further hinder generalization and stakeholder representation [19,75,79,121,156]. Studies focusing on only one or two communities (e.g., [28,57,92]) may reflect localized conditions, limiting their broader applicability [63].

The prevalence of qualitative methods underscores the need for quantitative approaches, such as food price surveys, cross-community comparative studies, and regression models, to validate and extend findings [5]. The prevalence of cross-sectional designs also constrains insight into long-term dynamics, highlighting the need for longitudinal studies [56,155]. Other methodological challenges include incomplete food store databases [93], limited price transparency in small outlets [94], and reliance on self-reported stakeholder data [50]. Seasonal fluctuations further complicate data collection, as certain foods are not available year-round [92]. Transportation costs and reliability, cultural practices, and infrastructure disruptions are often overlooked in PFSC models in these settings [14]. While advanced methods, such as agent-based modeling and stochastic optimization, can address these complexities, their application in remote Indigenous PFSC contexts remains theoretical mainly (e.g., [131]). Despite these advancements, the literature remains unclear in addressing accessibility challenges within remote PFSC and often fails to incorporate critical contextual factors [157]. Third, most studies focus narrowly on fruits and vegetables as indicators of healthy food access, neglecting other essential food groups such as whole grains and lean proteins [6,66]. Fourth, the geographic scope of current research is heavily skewed toward high-income countries, notably Australia, the United States, and Canada, leaving remote Indigenous communities in middle- and low-income countries significantly underrepresented [23,32]. This gap is concerning, as these communities often face similar or more severe PFSCM challenges while simultaneously developing innovative, culturally grounded solutions. Documenting and learning from these context-specific strategies could inform more adaptable and inclusive frameworks for improving PFSCM across diverse Indigenous settings [32].

## 6. Conclusions

This scoping literature review mapped and synthesized 84 studies from 14 countries to identify the challenges and practices of managing PFSC in remote Indigenous communities. By systematically mapping evidence across all levels of the supply chain, from production and distribution to retail and consumption, the review highlights how PFSCM challenges are associated with multiple dimensions of food access, including quantity, affordability, quality, safety, variety, and cultural acceptability. Building on this synthesis, the review identifies key PFSCM practices reported in the literature, including PFSC redesign strategies, forecasting and decision-support models, technological approaches, collaboration and coordination strategies, and targeted investments. These practices are integrated into a comprehensive conceptual framework encompassing seven interconnected PFSCM clusters: the PFSC system, decision-making, infrastructure and industry, risk factors, information management, technological advancements, and investment and capacity building. The framework is presented as an integrative, theory-informed sense-making structure that organizes patterns, interdependencies, and thematic linkages identified across the reviewed studies. By mapping existing evidence, synthesizing reported practices, and developing a conceptual framework, this review advances both theory and practice, providing a foundation for sustainable, context-sensitive interventions that enhance food system resilience and health equity in remote Indigenous communities.

The theoretical contributions of this review are fourfold. First, it addresses gaps identified by [23,31,32,33] by offering a multi-scalar, system-oriented synthesis of PFSCM literature, highlighting how supply chain challenges and practices are discussed across interconnected levels. Second, by shifting the analytical focus from food availability to the broader dimensions of food access, the review clarifies how PFSCM is framed in relation to food security in remote Indigenous contexts. Third, it expands the theoretical scope of PFSCM research by integrating evidence from high-, middle-, and low-income countries, addressing global imbalances in the literature, and generating comparative insights that enrich the development of cross-contextual theory. Fourth, this scoping review contributes an integrative conceptual framework that brings together dispersed evidence by systematically linking reported PFSCM practices, thematic clusters, and multiple food access components. By structuring how challenges and practices are discussed across supply chain levels, the framework clarifies complex interdependencies in the literature and provides a coherent analytical basis for comparison, synthesis, and future empirical inquiry.

From a managerial and policy perspective, the review highlights themes in the literature on decentralized, community-driven supply chain arrangements and the need to foster collaboration among local growers, suppliers, distributors, retailers, and community stakeholders. The review also underscores the limitations of isolated policy interventions, such as subsidies, without integrated system design, capacity building, and culturally relevant planning. The findings of this review are intended to inform policymakers, regulators, PFSC stakeholders, and food market analysts by providing a synthesized, evidence-informed overview of PFSCM challenges and practices, thereby supporting context-sensitive reflection, dialogue, and future decision-making related to perishable food access in remote Indigenous communities. This research has three main limitations. First, the review is limited to publications in English, which may have led to the omission of relevant studies in other languages. Second, although this scoping review included grey literature, it did not provide a thorough evaluation of the methodological quality of the included studies, which may have affected the reliability and validity of the results. Third, the scope of this review was confined to perishable foods available through market-based systems, thereby excluding traditional or subsistence foods exchanged outside formal markets.

## Figures and Tables

**Table 1:** Context-specific distribution-level challenges and their impacts on food access in remote Indigenous communities.

Distribution-Related Challenges	Context	Food Access Components					Sources
		Quantity	Affordability	Cultural Acceptability	Variety	Quality	
Long-distance transport	Canada (BC, Alberta, Manitoba, Ontario), Australia, Greenland	✓	✓	×	✓	✓	[9,21,53,58,74,81,84]
No year-round road access	Canada (subarctic Ontario), Australia, Greenland	✓	✓	×	×	×	[5,28,58,78,85]
Centralized distribution hubs	Rural Queensland (Australia)	✓	✓	×	×	×	[21]
Multi-stop air deliveries	Remote Alaska, Canada (Inuit communities)	×	×	×	×	✓	[75,86]
Unpredictable weather	Canada (Arctic), Australia (Northern Territory), Greenland	✓	✓	×	×	✓	[9,28,79,86]
High fuel costs	Northern Canada (Manitoba), Alaska, Australia	✓	✓	×	✓	×	[5,87]
Unflexible transportation mode	Remote communities in Western Australia, Remote Inuit communities, and communities in the Yukon-Kuskokwim Region of rural Alaska	✓	✓	×	✓	✓	[9,79,86,87]
Lack of cold chain infrastructure	Australia (Aboriginal communities), Northern Canada	✓	✓	×	×	✓	[13,33,77,87]
Vendor minimum orders	Navajo Nation (USA), Alaska	✓	✓	✓	✓	×	[19,86]
Infrequent deliveries	Greenland, Alaska, Canada	✓	×	×	✓	✓	[9,28]

**Table 2:** Context-specific retail-level challenges and their impacts on food access components in remote Indigenous communities.

Challenges	Context	Food Access Components					Sources
		Quantity	Affordability	Cultural Acceptability	Variety	Quality	
Limited competition in retail food markets	Remote Indigenous areas of Canada (e.g., Wapekeka in Ontario) and Western Australia	×	✓	×	✓	✓	[5,9,33,74,90,91]
Store type and food access	Subarctic Ontario (Canada), Gitxaala (British Columbia, Canada), Navajo Nation, Guam, India	×	✓	✓	✓	✓	[58,65,91,94,95]
Inadequate store infrastructure	Canada and Australia	✓	×	×	×	✓	[9,79,87,96,97]
Fluctuating prices	Australia and Greenland	×	✓	×	✓	×	[28,100,101]
Demand-supply mismatches	Arctic Canada, Aboriginal & Torres Strait Islander communities (Australia)	×	✓	✓	×	✓	[9,78,87,90]

**Table 3:** Context-specific PFSC redesign strategies and their impacts on food access.

PFSC Redesign Strategies	Context	Food Access Components	References
Community-based solutions	Subarctic Ontario, Nunavik, Nunavut, Saskatchewan, Alaska	Cultural acceptability, availability, affordability, and variety	[52,55,58,60]
Multi-echelon distribution hubs	Remote rural areas of Canada	Accessibility, affordability	[109,110]
Drone logistics hubs	Remote rural regions of Canada	Accessibility	[111]
Decentralized delivery systems	U.S. Indigenous communities, rural French Polynesia	Accessibility, cultural acceptability	[59,73,82]
Proximity-based retail models	Urban Indigenous communities, remote Australia	Accessibility, affordability	[9,59]

**Table 4:** Optimization models in the remote supply chain.

Author	Deterministic/Stochastic	Decision	What Is Included
[117]	Deterministic	Road upgrade	Coverage/costs
[109]	Deterministic	Hub allocation	Profits
[111]	Deterministic	Hub allocation	Distance
[120]	Deterministic	Food logistics components (collection, sorting, baling)	Not specified
[119]	Deterministic	Pilers	Cost
[110]	Deterministic	Hub allocation	Accessibility
[118]	Deterministic	Location-allocation-routing for food banks	Food access

## Data Availability

The authors confirm that the data supporting the findings of this study are available within the article and/or its Supplementary Materials.

## Supplementary Materials

The following supporting information can be downloaded at: https://doi.org/10.5281/zenodo.18270378, Table S1: Preferred Reporting Items for Systematic reviews and Meta-Analyses extension for Scoping Reviews (PRISMA-ScR) Checklist, Table S2: PCC Model, Table S3: Eligibility criteria for screening, Table S4: Literature search keywords, Table S5: Pilot database search results, Table S6: Data extraction questionnaire.

## Abbreviations

The following abbreviations are used in this manuscript:

PFSC	Perishable food supply chain
PFSCM	Perishable food supply chain management
PCC	Population/Phenomenon, Concept, and Context
ICTs	Information and Communication Technologies
PRISMA	Preferred Reporting Items for Systematic Reviews and Meta-Analyses
PRISMA-ScR	Preferred Reporting Items for Systematic Reviews and Meta-Analyses Extension for Scoping Reviews
JBI	Joanna Briggs Institute
SCM	Supply Chain Management
FSC	Food Supply Chain
FAO	Food and Agriculture Organization of the United Nations
NNC	Nutrition North Canada
GST	Goods and Services Tax
